# Stepping stones to extirpation: Puma patch occupancy thresholds in an urban‐wildland matrix

**DOI:** 10.1002/ece3.10381

**Published:** 2023-08-04

**Authors:** David C. Stoner, Zara McDonald, Courtney A. C. Coon

**Affiliations:** ^1^ Department of Wildland Resources Utah State University Logan Utah USA; ^2^ Felidae Conservation Fund Mill Valley California USA

**Keywords:** California, connectivity, cougar, extirpation, fragmentation, occupancy, *Puma concolor*, threshold, urban‐wildland interface

## Abstract

Habitat loss and fragmentation are the leading causes of species range contraction and extirpation, worldwide. Factors that predict sensitivity to fragmentation include high trophic level, large body size, and extensive spatial requirements. Pumas (*Puma concolor*) exemplify these qualities, making them particularly susceptible to fragmentation and subsequent reductions in demographic connectivity. The chaparral‐dominated ecosystems surrounding the greater San Francisco Bay Area encompass over 10,000 km^2^ of suitable puma habitat, but inland waterways, croplands, urban land uses, and extensive transportation infrastructure have resulted in widespread habitat fragmentation. Pumas in this region now exist as a metapopulation marked by loss of genetic diversity, collisions with vehicles, and extensive human–puma conflict. Given these trends, we conducted a photo survey from 2017 to 2021 across 19 patches of predicted habitat and compiled a dataset of >6584 puma images. We used a logistic regression analytical framework to evaluate the hypothesis that puma patch occupancy would exhibit a threshold response explained by patch size, isolation, and habitat quality. Contrary to predictions, only variables related to patch size demonstrated any power to explain occupancy. On average, occupied patches were 18× larger than those where they were not detected (825 ± 1238 vs. 46 ± 101 km^2^). Although we observed pumas in patches as small as 1 km^2^, logistic regression models indicated a threshold occupancy probability between 300 and 400 km^2^, which is remarkably close to the mean male puma home range size in coastal California (~381 km^2^). Puma populations dependent on habitats below this value may be susceptible to inbreeding depression and human–wildlife conflict, and therefore vulnerable to extirpation. For species conservation, we suggest conflicts might be ameliorated by identifying the largest, isolated patches for public education campaigns with respect to management of domestic animals, and remaining connective parcels be identified, mapped, and prioritized for targeted mitigation.

## Graphical abstract

This manuscript details efforts to estimate thresholds in habitat patch size predicting the occupancy of pumas (*Puma concolor*) in a highly fragmented ecosystem around the San Francisco Bay area. We argue that the empirical threshold identified herein corresponds to the mean male home range size of a puma, as measured in the chaparral‐dominated ecosystems of coastal California. Furthermore, this metric may be useful for identifying subpopulations at risk of extirpation. Because of the combined concern over human safety and species conservation the topic is of interest to policy makers and wildlife managers in California and other western jurisdictions.

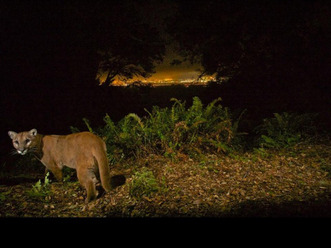

## 1 INTRODUCTION

Loss of demographic connectivity is a leading cause of species range contractions and extirpations, worldwide (Crooks et al., 2017; Jacobson et al., 2019; Semper‐Pascual et al., 2021). Expansion of agriculture, urban land uses, and transportation infrastructure results in fragmentation, systematically reducing patch size, and increasing isolation and edge‐area ratios. Anthropogenic barriers such as dams, fencing, or highways, can reduce or eliminate demographic connectivity in aquatic and terrestrial systems (Marschall et al., 2011; Seidler et al., 2014). High edge‐area ratios in occupied habitat can simultaneously facilitate the spread of invasive species (Haddad et al., 2015) and promote human–wildlife conflict (Woodroffe & Ginsberg, 1998). These patterns interact to reduce the suitability of remaining habitats or isolate them altogether, resulting in non‐linear trends in the rate of habitat loss and therefore community composition (Wilson et al., 2016). Yet, despite the ubiquity and acceleration of this problem, few extirpation thresholds of habitat area have been estimated for species of conservation concern.

Factors predicting vulnerability to extirpation have been studied extensively. Life history traits, niche specialization, and trophic level all contribute (Davidson et al., 2009), but isolation and habitat area have proven strong predictors of population persistence (Crooks et al., 2011; González‐Suárez & Revilla, 2014). The effects of these factors are scale‐sensitive, with large‐bodied, obligate carnivores most likely to be impacted by current land‐use trends because of extensive spatial requirements and low population densities (Stoner et al., 2018). Therefore, species at high trophic levels come into contact with hard boundaries at greater frequencies than those with smaller home ranges and resource needs (Woodroffe & Ginsberg, 1998). As such, retaining or restoring demographic connectivity among sub‐populations has become one of the prevailing themes in wildlife management, with major conservation efforts focused on carnivores and/or migratory ungulates (e.g., USDI, 2018).

**FIGURE 1:**
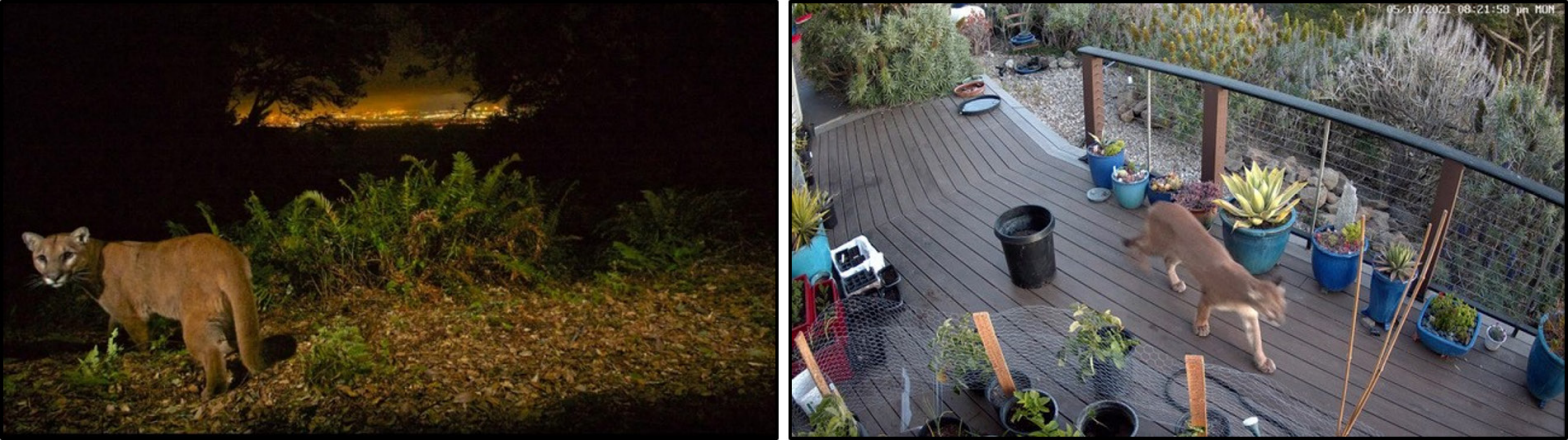
Pumas occupy wildlands adjacent to major urban areas, but also traverse developed landscapes where they are vulnerable to various forms of human–wildlife conflict (photos courtesy of Steve Winter and Andy Forward).

Pumas (*Puma concolor*) are one of the most broadly distributed mammals in the western hemisphere, inhabiting a wide variety of climatic zones and land‐use types, including near‐urban environments (Blecha et al., 2016, Riley et al., 2021, Stoner et al., 2021; Figure 1). As large‐bodied felids, pumas are strict carnivores that depend on the presence and abundance of ungulate prey to support them (Pierce & Bleich, 2003). They occupy the highest trophic level, making them numerically rare in comparison with similar‐sized omnivores and herbivores in the same systems. Although buffered from extinction by their large geographic range and ecological tolerance (Culver et al., 2000), pumas exemplify many of the qualities that make a species vulnerable to extirpation at local scales (Davidson et al., 2009; Purvis et al., 2000; Stoner et al., 2018).

Scientific investigations of pumas have occurred throughout the species' North American range. Despite exceptional dispersal abilities (Hawley et al., 2016; Stoner et al., 2013), research indicates that when combined with natural habitat patchiness, anthropogenic features can amplify fragmentation, thereby constraining puma movements (Stoner et al., 2013), or isolating subpopulations (Benson et al., 2019; Maehr et al., 2002). Some of the most compelling examples of this come from California and Florida (e.g., Beier, 1995; Maehr et al., 2002). The Florida panther (*Puma concolor coryi*) exists as a single, relict population with no connectivity to other extant populations, whereas California represents a bellwether for the effects of habitat fragmentation on species demographic connectivity (Benson et al., 2019; Dellinger et al., 2020). Indeed, several California puma populations number fewer than 100 individuals (e.g., Beier, 1993; Benson et al., 2020), and thus occur as sub‐populations within a metapopulation context (Beier, 1993; Sweanor et al., 2000). Small and isolated populations are vulnerable to extirpation through stochastic events (Benson et al., 2019; Choate et al., 2018; Crooks, 2002; van de Kerk et al., 2019) or chronic anthropogenic stressors such as poisons, vehicle accidents, or depredation removals (Benson et al., 2020; Stoner et al., 2021). Under these conditions retaining connectivity among smaller patches is critical for population persistence (Suraci et al., 2020).

There is a growing concern among state wildlife agencies over the conservation value of small habitat patches (Fahrig et al., 2022), but as yet, there are no estimates of threshold values of habitat area to proactively identify populations at risk. Dellinger et al. (2020) calculated that 8000–15,000 km^2^ of connected habitat were required to maintain an effective population size of 50 adult pumas and therefore genetic integrity. These authors identified five populations in California that did not meet this threshold, prompting policies designed to provide greater protections for those subpopulations vulnerable to inbreeding and subsequent declines or extirpation. Based on these trends, we set out to test the hypothesis that puma patch occupancy, and by proxy, local range contractions, would exhibit a threshold response to habitat fragmentation (Crooks, 2002). We predicted that habitat patch size, isolation, and quality would best explain trends in puma presence (sensu MacArthur & Wilson, 1967), and that this would be influenced by patterns in land use and landcover. Furthermore, we predicted that area thresholds would correlate with puma spatial requirements typical for local environmental conditions.

## 2 MATERIALS AND METHODS

### 2.1 Study area

**FIGURE 2:**
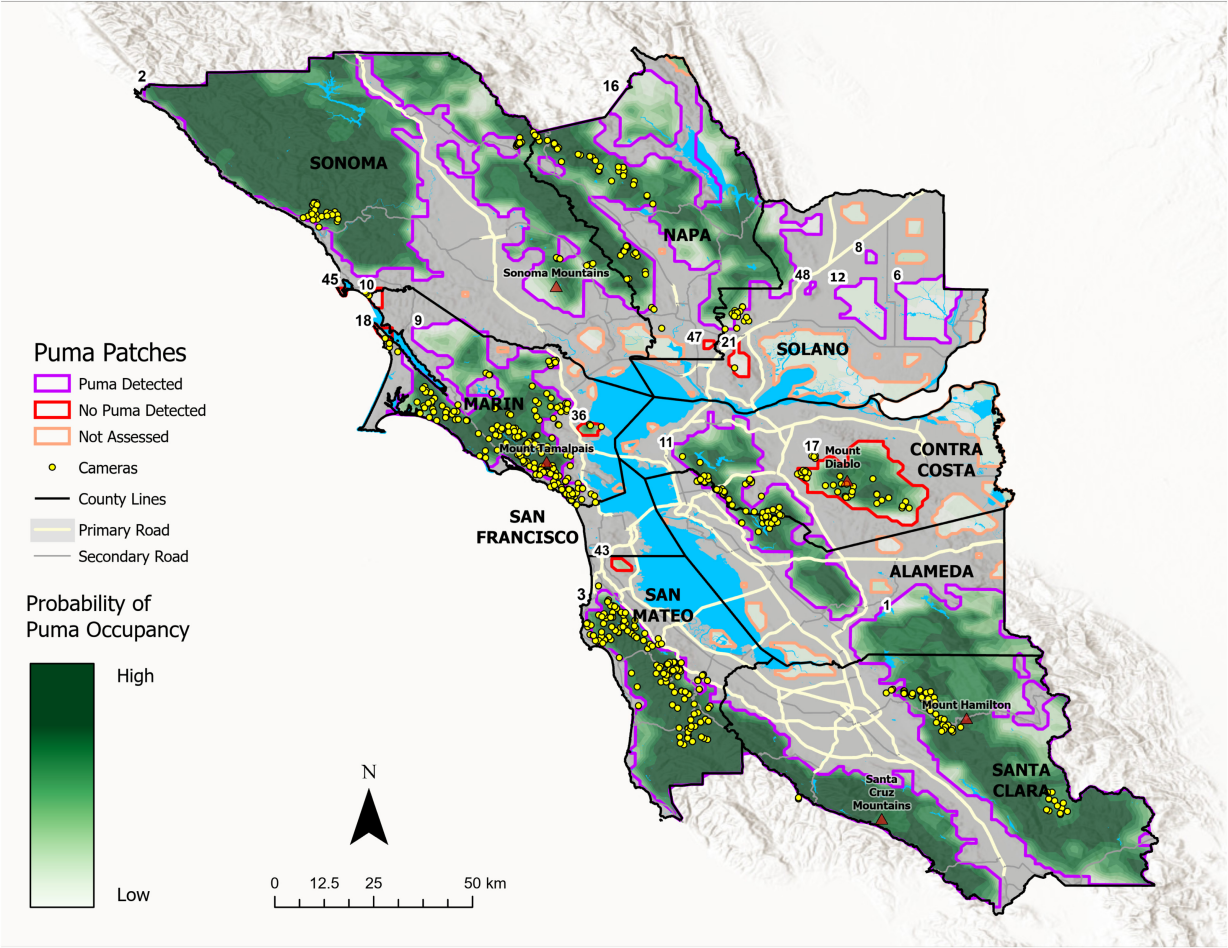
Outline of nine San Francisco Bay Area counties in California that constitute the study area. Color‐coded habitat patches are based on (1) whether the patch was enrolled in the study, and (2) whether or not a puma was detected during the sampling period (2017–2021).

To evaluate this hypothesis, we estimated puma patch occupancy across the nine counties that comprise the greater San Francisco Bay Area in northern California, USA. The region measures 18,152 km^2^, and includes Sonoma, Napa, Marin and Solano Counties in the north, Contra Costa and Alameda Counties in the east, and San Francisco, San Mateo, and Santa Clara Counties in the south (Figure 2). Based on U.S. Census Bureau data published in 2020, the population of these counties was approximately 7.5 million people at the time of the study (https://www.census.gov/). Land uses range from minimally disturbed wildlands to dense urban areas, with suburban, exurban, agricultural, industrial areas, and open spaces comprising the gradient between wilderness and urban landscapes. The climate is Mediterranean, defined by warm, dry summers (10–33°C) and cool, rainy winters (2–18°C; 500–1200 mm precipitation; https://www.usclimatedata.com/). Local climate varies spatially as a function of elevation and distance from the ocean. The marine fog belt maintains cooler and more consistent temperatures along the coast as compared to inland sites at the same elevation and latitude. Dominant plant communities reflect this climatic regime, and vary from chaparral shrublands near the coast, which grade into mixed oak woodlands and grasslands further inland. The area exhibits a high degree of natural fragmentation stemming from waterways that comprise the San Francisco Bay, Suisun Bay, and the Sacramento Delta. Anthropogenic land uses are draped over these drainage patterns and serve to both amplify fragmentation and attract wildlife (Coon et al., 2020).

### 2.2 Study patches

To identify habitat patches for field sampling, we used predictions of suitable habitat from Coon et al. (2020). This model used 4 years of camera trap data to predict occupancy probabilities for every 1 km^2^ of the study area during both wet (winter) and dry (summer) seasons. The model was built with positive occupancy estimates for both forest cover (including evergreen, mixed, and deciduous forests) and distance to roads. For the current analysis, we sampled study patches from the dry season model by selecting the 1 km^2^ pixels with the highest 50% real and extrapolated occupancy probabilities, and then grouped all 1 km^2^ pixels adjacent to at least one other top‐50% patch. Predicted occupancy patches for pumas were larger in the dry season, and therefore we used this delineation to draw habitat fragment boundaries. The model produced occupancy predictions for 51 individual patches in the study area, ranging in size from 1 to 4000 km^2^. However, 32 of these patches were eliminated from the sample frame due to inaccessibility (private or military lands), or resource limitations which precluded sampling of the smallest, most isolated patches or those dominated by wetlands with little structural vegetative cover (Figure 2).

### 2.3 Puma occupancy

Our response variable was puma presence (or detection) within a given habitat patch, which we used as an index of occupancy. We did not use formal occupancy models per se (sensu MacKenzie et al., 2003), but used the term in the vernacular, meaning *occurring in a place*. To measure this, we sampled photos from a master database of more than 329,000 images derived from 483 remote camera placements conducted from 2017 to 2021. Trail camera images were cataloged by project biologists and volunteers, and candidate puma photos were validated by the author (CACC). From this dataset, we compiled 6584 images of pumas from across the study area. Within selected habitat patches, initial camera deployments were placed strategically to maximize the probability of detecting wildlife on public lands and sites where we had permission to access private lands. Our criteria included the following: cameras were always ≥2 km apart, using common trigger speeds and motion sensors to maintain consistency; and no scents or lures were used to repel or attract wildlife. Camera deployment length varied by site, and as such, there was no maximum length of deployment. Instead, we defined a minimum threshold for justifying puma absence. Camera stations were established at densities of approximately 1 camera per 5 km^2^ (0.2 cameras per km^2^), and were active for 1–1974 days (mean = 214, SD = 269 days). Within this dataset, 180 camera placements had one or more puma detections and 94 had at least two detections (mean number detections at cameras with ≥1 detection = 11.2, SD = 26.7). Puma occupancy of a patch was determined through collection of at least one piece of verifiable evidence. These data came primarily from the long‐term camera survey conducted by our non‐profit organization (Felidae Conservation Fund, Bay Area Puma Project), and were supplemented with images provided by colleagues at state, county, and non‐profit agencies, photos with geotags submitted by email or to our organizational citizen scientist online database (www.BAPP.org/sightings‐map), and publicly available observations of pumas marked by other organizations coincident with our camera survey (2016–2020; Table 2). Detections were not treated as independent.

Presence/absence surveys are prone to false negatives, in which putative absences do not preclude the possibility that animals were present but went undetected during the sampling interval. To derive a rule set for determining absence, we used data from cameras that were in the field for at least 115 days and produced one or more puma detections. Preliminary calculations indicated 90% of confirmed detections occurred within the first 124 days of sampling. Additionally, at camera sites in which a puma was detected on at least two occasions, mean latency between detections was 80 days. We averaged these two values to set a minimum density and sampling duration to evaluate presumed absences. Specifically, we multiplied 0.2 cameras/km^2^ by 102 days to get a minimum required 20.4 camera‐days/km^2^ in each patch. Although false negatives were still possible, this criterion provided a consistent, repeatable standard for justifying puma absence from a given habitat patch.

### 2.4 Predictor variables

To assess how patch size affected patterns in puma occupancy, we tested three variables describing different aspects of area, including patch size (km^2^), perimeter (km), and the perimeter–area ratio. All were calculated using ArcGIS V. 10.7 software (Environmental Systems Research Institute). We also tested secondary variables hypothesized to affect patch occupancy. These included “isolation,” defined here as the distance between patches weighted by patch size (MacGarigal & Marks, 1995; Table 1); and “naturalness”—a continuous index of habitat quality that uses landcover, housing density, road presence, and traffic volume to scale anthropogenic disturbance (Theobold et al., 2012). Isolation and naturalness were calculated for each patch and an accompanying 1 km buffer. The naturalness buffer was used as a proxy for ecotones, that is, forest–grassland edges, which provide foraging habitat for mule deer (*Odocoileus hemionus*), and stalking cover for pumas (Holmes & Laundre, 2006). We then calculated the percent of landcover types that influence puma occupancy and prey vulnerability, including grassland/agriculture, and forest cover (Coon et al., 2020). Lastly, we evaluated several variables that have particular relevance in a study area characterized by extensive habitat alterations, such as non‐native vegetation and impermeable surfaces. These included land ownership (% private), percent freshwater, percent developed, and relative road length. All variable definitions, units, and sources are detailed in Table 1.

**TABLE 1:** List of variables, measurement units, source data, transformations, and *t*‐test results for factors hypothesized to explain puma patch occupancy (San Francisco Bay Area, CA, 2017–2021).

Variable	Notes	Unit	Data source	Transformation	*t*	95% CI	df	*p*
Patch size	–	km^2^	ArcGIS	Log	−2.29	−4.41, −0.16	15.5	.04
Patch perimeter	–	km	ArcGIS	Log	−2.51	−2.79, −0.22	14.5	.03
Perimeter–area ratio	–	NA	ArcGIS	Log	1.88	−0.10, 1.66	16.6	.08
Private‐ownership	–	%	BLM^a^ & GreenInfo^b^	Arcsine‐square‐root	−0.11	−0.41, 0.37	16.7	.91
Grassland/agriculture	–	%	USDA NASS^c^	Arcsine‐square‐root	−1.84	−0.65, 0.05	15.9	.09
Forest cover	Includes evergreen, mixed, deciduous	%	USDA NASS^c^	Arcsine‐square‐root	−0.09	−0.35, 0.32	16.4	.93
Fresh water	–	%	USDA NASS^c^	Arcsine‐square‐root	−0.98	−0.13, 0.5	16.2	.34
Medium to high development	Impervious surfaces account for 50–100 of total cover	%	USDA NASS^c^	Arcsine‐square‐root	1.59	−0.3, 0.17	8	.15
Relative road length	Summed total highways and roads that support vehicle traffic divided by patch area (km^2^)	NA	MTC^d^	Log	−0.60	−4.70, 2.64	13.7	.56
Averaged indices of naturalness (patch)	Average patch intactness within patch on a −1 (disturbed) to 1 (natural) scale	NA	CBI^e^	None	0.20	−0.37, 0.45	15.9	.84
Averaged indices of naturalness (buffer)	Average buffer intactness, on −1 to 1 scale, in a 1‐km buffer around each patch	NA	CBI^e^	None	0.50	−0.31, 0.50	14.2	.63
Isolation index	Index of patch proximity to other patches, accounting for size of nearest patches	NA	FRAGSTATS PROX^f^	Log	0.24	−1.33, 1.66	16.6	.82

^a^ Bureau of Land Management (BLM): https://navigator.blm.gov/data?id=1fca0357df7c87ae.

^b^ GreenInfo Network: https://www.calands.org/.

^c^ United States Department of Agriculture – National Agricultural Statistics Cropland Data Service (USDA NASS): https://nassgeodata.gmu.edu/CropScape/.

^d^ Metropolitan Transportation Commission (MTC): https://hub.arcgis.com/datasets/MTC::san‐francisco‐bay‐region‐roadways/about.

^e^ Conservation Biology Institute (CBI): https://databasin.org/datasets/e3ee00e8d94a4de58082fdbc91248a65.

^f^ FRAGSTATS PROX tool: https://www.umass.edu/landeco/research/fragstats/documents/Metrics/Isolation%20‐%20Proximity%20Metrics/FRAGSTATS%20Metrics.html.

### 2.5 Analyses

We used Welch's two‐sample *t*‐tests to assess the significance of individual predictor variables on puma detection. *T*‐tests were run with the t.test() function from the Stats package in program R (R Core Team, 2013). We then built a global multiple logistic regression model using variables with significant and marginally significant *t*‐test results, while accounting for correlation between variables. Logistic regression models were analyzed with the generalized linear model (glm()), also from the Stats package, using a binomial distribution and a logit link, with puma presence (1) or absence (0) as the response variable. The global model was used to create a ranked model selection table with the dredge() function. We then used the importance() function to determine variable importance on a 0–1 scale for each predictor in the global model; both functions are part of the MuMIn package. To identify potential area thresholds in occupancy, we built a simple logistic regression model using patch size (log km^2^) as the single predictor. Where appropriate, variables were transformed to meet assumptions of normality (Table 1).

## 3 RESULTS

Of the 51 candidate patches identified from the puma habitat model, we were able to conduct field surveys, or obtain photographic evidence from 19 patches. Eleven patches produced evidence of being used by at least one puma during the sampling interval (Figure 2, Table 2). Eight sites were surveyed for the minimum sampling interval, but produced no detections (Appendix 1). Ideally, cameras would be set up at 1 unit per 5 km^2^ (0.2) but because of the mosaic of ownership within patches, it was not always possible to systematically sample entire patches, hence the reason we report camera numbers and densities within study patches (Appendix 1). At a camera density of 0.2 cameras/km^2^ for 102 days, we required a minimum of 20.4 (camera‐days)/km^2^ to confirm the absence of pumas in the study patch, which was met for all 19 focal patches.

**TABLE 2:** Summary of the 19 patches surveyed and their size, perimeter, and detection status in the San Francisco Bay Area, CA (2017–2021).

Map ID	Patch name	Size (km^2^)	Perimeter (km)	Naturalness	Intactness	No. cameras	No. puma detections	Detection source
2	Sonoma Napa/Solano	4022	822	Very low	0.48 (mod low)	7	607	Multiple^a^
1	Santa Clara/Henry Coe State Park	1969	353	Very low	0.72 (mod high)	12	123	Multiple^a^
3	San Mateo/Santa Cruz Mtns	1353	354	Low	0.34 (mod low)	49	5764	Multiple^a^
9	Marin/Sonoma	731	260	High	0.53 (mod high)	37	83	Multiple^a^
11	Tilden, Sibley, Chabot Co. Parks	583	191	Very low	−0.16 (low)	0	2	Citizen^c^
17	Mt Diablo State Park	295	85	Moderate	0.31 (mod low)	10	0	
16	Berryessa Estates	189	61	Very low	0.78 (high)	1	1	Camera trap^c^
6	No. Rio Vista	141	56	High	−0.59 (mod low)	0	1	Collar data^b^
12	Travis Air Force Base	83	47	High	−0.20 (mod low)	0	1	Collar data^b^
21	NE Vallejo/Hiddenbrook	23	24	Very low	0.47 (mod high)	2	0	
36	China Camp	12	14	High	−0.45 (low)	0	0	
10	Estero Americano	11	14	Low	0.67 (high)	2	0	
43	San Bruno Mtn	10	12	High	−0.57 (low)	2	0	
18	No. Pt Reyes National Seashore	9	15	High	0.38 (mod high)	2	0	
8	NE Fairfield	6	9	High	−0.62 (low)	0	1	Collar data^b^
47	American Canyon (Newell OS)	4	7	Very low	0.39 (mod high)	3	0	
48	Vacaville	3	9	Moderate	−0.17 (mod low)	0	1	Collar data^b^
45	Sonoma Coast State Park	3	7	Very low	0.03 (mod low)	1	0	
42	Ano Nuevo State Park	1	4	Very low	−0.13 (mod low)	Unknown^d^	>1	Camera trap^a^

*Note*: Puma presence was confirmed on 11 patches. All camera‐based monitoring efforts met the minimum 6‐month criterion for sampling duration. For column 9 (detection source), details are as follows.

^a^ Confirmation of presence came from multiple sources which may have included Felidae camera trap data, colleagues' camera traps, or verifiable images shared by local citizens.

^b^ Collared puma data made publicly available by Audubon Canyon Ranch Lion Project.

^c^ Verifiable images/videos were collected and submitted by multiple individuals through Felidae's Puma and Bobcat Sightings Map (www.BAPP.org/sightings‐map).

^d^ An unknown number of camera traps were established and maintained by colleagues at Ano Nuevo State Park general wildlife monitoring purposes. Multiple photos were shared with us to confirm species identification and date.

**FIGURE 3:**
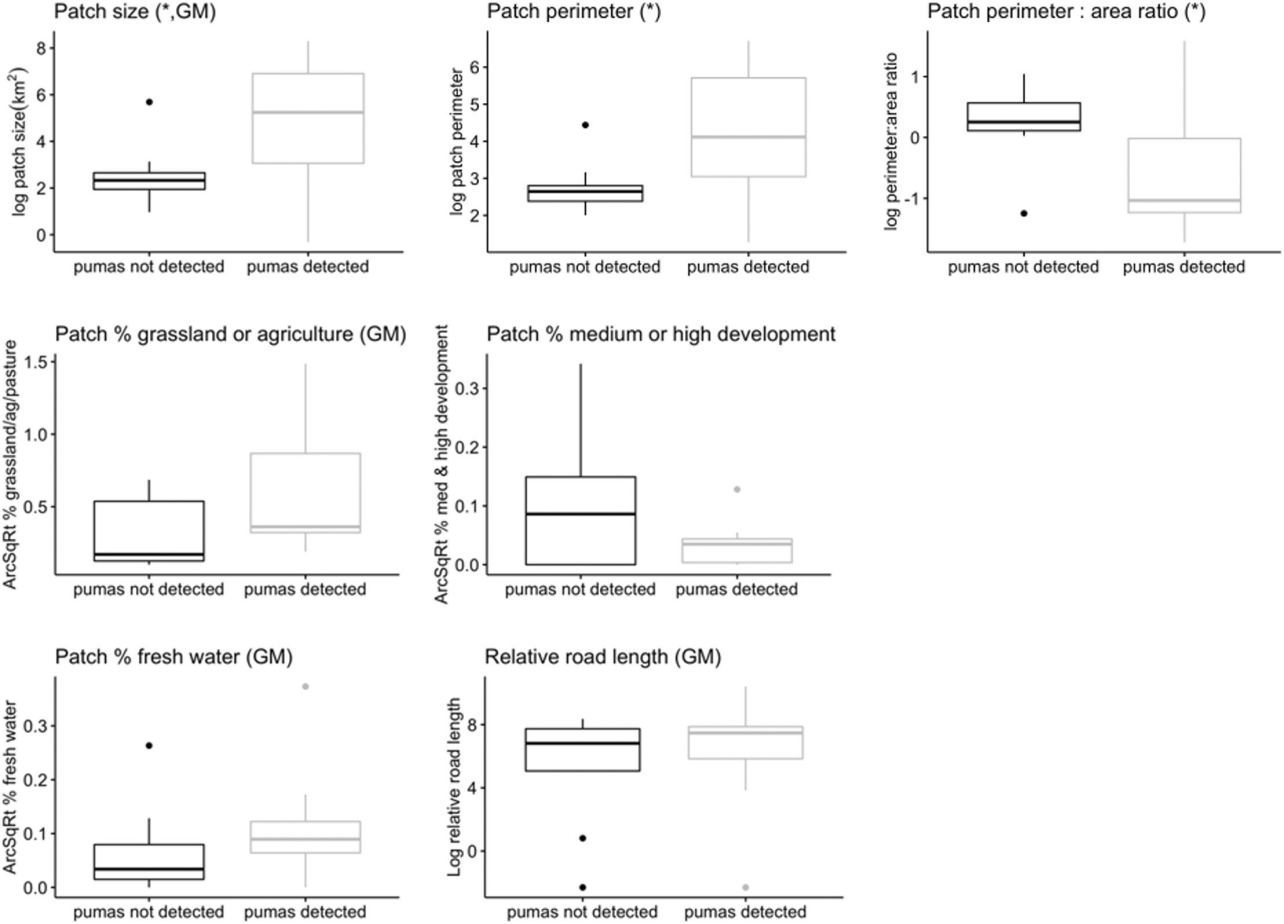
Box plots illustrating predictor variables with significant *t*‐test results (*) or variables included in the global binomial multiple regression model (GM), San Francisco Bay Area, CA (2017–2021).

Variables that provided the strongest evidence for differences in occupancy were mean patch size, patch perimeter length, perimeter–area ratio, percent grassland/agriculture, and percent anthropogenic development (*p* < .15; Table 1, Figure 3). We found no statistical differences between occupied and unoccupied patches with respect to isolation, naturalness, naturalness within a 1 km buffer, percent forest cover, percent private ownership, percent fresh water, and summed length of roads (*p* > .30; Table 1, Figure 3).

We used a combination of *t*‐test results and cases of multicollinearity to determine which variables to include in the global model. The global model included: patch size, patch perimeter, percent grassland/agriculture, percent development, percent fresh water, and road length within a patch, but we were unable to use all variables with significant or marginally significant *t*‐test results (*p* < .10) due to multicollinearity among predictors. For example, patch size, perimeter, and perimeter–area ratio all had significant *t*‐tests (Table 1), but those three variables and percent forested were all correlated (*r* ≈ .50; Appendix 2). Thus, for the global model we only included patch size, as it not only had a high *t*‐value and low *p*‐value (Table 1), but is also a standard metric for evaluating conservation value of a given parcel.

After model selection with dredge(), the most informative model included patch size (estimate = 0.50, SE = 0.27, *p* = .07), and percent anthropogenic development (*e* = −17.3, SE = 11.8, *p* = .14; model AIC = 23.8, AICc = 25.4). Because of concerns over the potential for residual collinearity to inflate the beta coefficient, we calculated McFadden's pseudo‐*R* ^2^ for the best model. The result was 0.31 suggesting the model was not likely over fit. Although the variable percent grassland/agriculture was not in the best model, as a sole predictor of puma detection, it did appear marginally important (estimate = 3.16, SE = 2.05, *p* = .12). When considering all models within two or four AICc points, it had a similar level of importance to percent anthropogenic development (Table 3; Appendix 3). Percent fresh water and road length within a patch were not important (Table 3).

**TABLE 3:** Values of variable importance on a 0 to 1 scale calculated from ranked models from all possible models generated by a global multiple logistic regression or some subset based on ΔAICc from the best model.

Model category	Patch size*	Percent development*	Percent grassland/agriculture	Percent fresh water	Relative road length
Models within 2 AICc of best (*n* = 4)	0.78	0.53	0.44	0.00	0.00
Models within 4 AICc of best (*n* = 10)	0.66	0.51	0.45	0.17	0.18
All possible models (*n* = 16)	0.61	0.51	0.46	0.26	0.25

*Note*: Variables included patch perimeter, patch development, and percent pasture and agriculture (denoted with an * above).

**FIGURE 4:**
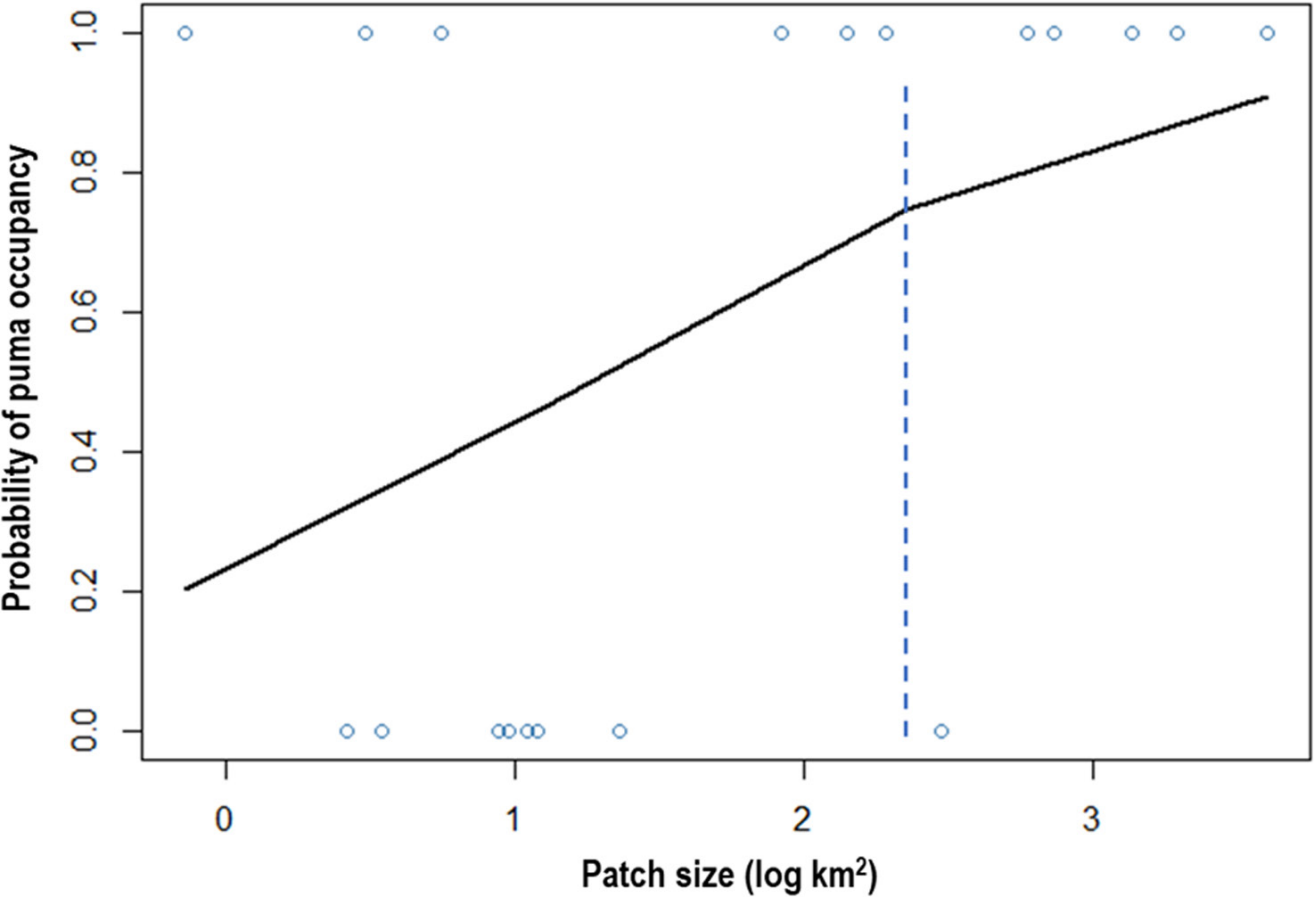
Logistic regression results illustrating puma detection probability as a function of patch size in the San Francisco, Bay Area, CA (2017–2021). Results suggest an occupancy threshold of approximately 300–400 km^2^ (dashed line).

Although we found evidence of pumas in patches as small as 1 km^2^, on average patches where puma presence was confirmed were 18× larger than those lacking detections (825 ± 1238 km^2^ vs. 46 ± 101 km^2^). Based on the simple logistic regression model with log (patch size) as the sole predictor, we identified a decrease in detection probability at patch sizes below log values of 2.5, or ~300–400 km^2^ (*n* = 19, *est.* = 0.43; *z* = 1.81; *p* = .07; Figure 4).

## 4 DISCUSSION

Our objective was to evaluate patterns in puma habitat patch occupancy in a region defined by extensive levels of natural and anthropogenic fragmentation. We defined occupancy broadly as presence within a patch under the assumption that detection of pumas connotes some conservation value, even if only used as a stepping stone within an array of larger patches (Beier, 1995; Lynch, 2019). As expected, variables related to patch size (area, perimeter length, and perimeter–area ratio) displayed consistently strong relationships with puma occupancy. Pumas were detected in patches as small as 1 km^2^, but logistic regression results indicated a threshold value of 300–400 km^2^, suggesting that patches below this size were unlikely to harbor pumas. In a similar analysis, Crooks (2002) reported that probability of puma detection was lowest in patches <15 km^2^, and highest in those exceeding 100 km^2^. The author made clear that population viability was questionable at the bottom end of those estimates. Landcover was largely uninformative, with pumas less likely to be detected in patches where buildings and pavement accounted for more than 50% of the area. However, this is likely an artifact of our sampling scheme, in which we used selection criteria that minimized variation in landcover characteristics, a priori. Patches with no puma detections were relatively small, sparsely vegetated, or dominated by urban land uses. Contrary to expectation, isolation and naturalness had no discernable effects on puma detection. This presents an apparent contradiction in the literature that is mirrored in our results: pumas prefer large, natural spaces and tend to avoid humans even in highly populated areas (Benson et al., 2016), yet they persist where prey resources are available, regardless of patch naturalness.

We delineated habitat patches based on the model presented in Coon et al. (2020), in which forest cover and road infrastructure were the strongest positive and negative predictors of habitat quality, respectively. At the scale of the individual patch neither of these variables were important in predicting puma detection. We suspect the lack of a relationship is related more to sampling criteria than to any inherent behavioral tendencies expressed by pumas. Patches targeted for sampling met some minimum values with respect to roads and forest cover, and as such, did not capture the full range of variation that exists across the greater study area. Moreover, the occupancy model used to create the patches in this study (Coon et al., 2020) was restricted to the 9‐county Bay Area, and as such, some of the edge patches based on county boundaries may be larger than estimated here.

The only landcover variable that positively impacted occupancy was the percentage of open habitat, such as grassland, pasture, or cultivated land. Puma habitat models consistently identify open, flat, or sparsely vegetated habitats as underused relative to availability, presumably because these cover types are incompatible with their stalk‐and‐pounce hunting style (Dickson & Beier, 2002; Logan & Irwin, 1985; Smereka et al., 2020). Our results largely support this generality, yet these same cover types and associated edges are preferred by black‐tailed deer (*Odocoileus hemionus columbianus*), the primary puma prey species in this region (Allen, 2014; Hopkins, 1989), as well as various synanthropic species (Bateman & Fleming, 2012). Pumas are successful at hunting in forest–grassland ecotones (Holmes & Laundre, 2006), and therefore the weak but positive correlation between puma presence and open habitat may be an optimization of these constraints.

Beyond size and isolation, tremendous variation exists among habitat patches within our sample. A surprising mismatch between model expectations and empirical confirmation is Mt Diablo State Park in Contra Costa County. This patch is exemplary in that, despite large size (~300 km^2^), protected status, extensive forest cover, and photographic confirmation of ungulate prey, we did not obtain a single puma photograph over the >6‐month sampling interval (4860 trap nights). Notably, based on a sample of 69 radio‐marked pumas, Suraci et al. (2020) reported zero successful dispersal movements to the Diablo Range or any other large patches neighboring the Santa Cruz Mountains. Although Mt Diablo is the single largest insular habitat patch within the 18,000 km^2^ study region, it is bounded on the west by the San Francisco Bay, on the north by the Sacramento Delta, to the south by a 12‐lane interstate (I‐580), and on the east by the extensive agricultural lands of the Sacramento Valley. Taken together, this suggests that this patch may already be sufficiently isolated to reduce immigration and may therefore be vulnerable to extirpation.

Pumas have been documented traveling through residential and urban environments (Riley et al., 2021; Suraci et al., 2020), but there are no examples of them occupying these areas indefinitely (Beier et al., 2010). Thus, the question still remains as to how animals are moving among the more isolated patches, given that indices of isolation had no effect on puma detection. Two recent analyses may provide some insights to this question. Suraci et al. (2020) studied pumas in the south‐western portion of the study region and suggested micro‐scale movement decisions based largely on attraction to vegetative cover and avoidance of urban landcover types. Other models of mammalian navigation suggest that in areas of high relief, transient animals may survey areas within line‐of‐sight prior to making extensive dispersal or migratory movements (Berger et al., 2022; Sweanor et al., 2000). Taken together, pumas negotiating fragmented environments may be using a combination of sensory cues, from immediate information about cover and prey availability, to directed movements based on long‐distance observations of landmarks correlated with suitable habitat. Absent impenetrable barriers, this suggests green space and residual riparian strips may serve as movement corridors in otherwise anthropogenically altered environments (McClanahan et al., 2017).

Although results are consistent with our primary hypothesis, all of this raises questions about the residency status of the animals observed during this survey. The pumas we detected on smaller patches may have used them for any of the following reasons: (1) small patches represent areas of high prey concentration and/or vulnerability, (2) individual pumas constructed temporary or permanent home ranges by using multiple small patches, or (3) dispersing animals used small patches as stepping stones to access larger habitats. Detection of a puma within a patch does not provide information about the actual value of that location to an individual, nor does it give any indication of population status. Pumas are not uniquely marked, thereby making our sampling methods insensitive to mark‐resight analyses. As such, with the exception of family groups, we could not systematically discern residents from transients. This handicap limits inference about population viability to crude measures of patch size.

Beier (1996) estimated that individual patches of 1000–2200 km^2^ would secure viability of a subpopulation at multi‐decadal scales. More recently, calculations by Dellinger et al. (2020) suggested that 10,000 km^2^ of contiguous habitat would be required to maintain an effective population size of 50 adult pumas to mitigate the effects of inbreeding. Puma social organization is characterized by a resident male overlapping two to five often‐related adult females (Logan & Sweanor, 2010). In the Mediterranean climates of coastal California puma home range size varies widely, but averages 153 and 381 km^2^ for females and males, respectively (Allen, 2014; Dickson & Beier, 2002; Hopkins, 1989). The male home range value is remarkably consistent with our threshold estimate. If we use this as a minimum demographic unit for conservation, then only 28% of surveyed patches meet this areal criterion. Using female home range under the assumption that males can travel among patches, then this number increases to 39% of patches. Yet, beyond the large, intact blocks of habitat marking the northern and southern edges of the study region (Figure 2), none of the individual patches with detections meet the criteria advanced in either of the aforementioned studies, suggesting that relatively smaller patches in this system may primarily function to promote emigration and gene flow between remaining large patches. Indeed, reproductive success and subsequent dispersal from large habitat blocks may be sustaining the occupancy of smaller, isolated patches that retain some suitable habitat (i.e., the source population concept; Cooley et al., 2009). However, it is unclear whether young, transient pumas create temporary home ranges from a collection of patches that individually are too small to sustain indefinite occupation, or if these patches may simply be functioning as stepping stones for animals dispersing from the Sonoma and Diablo Mountain Ranges through the urban‐wildland matrix. Taken together, our results suggest that the mosaic of occupied patches identified here may function as a metapopulation, in which individual demographic units go through phases of extirpation and recolonization (e.g., Beier, 1996; Benson et al., 2019).

## 5 MANAGEMENT IMPLICATIONS

The genetic diversity of some puma subpopulations in coastal California is nearly as low as the federally endangered Florida panther (*P. c. coryi*), which has raised concerns about long‐term persistence (California Department of Fish and Wildlife, 2018; Gustafson et al., 2018). Three aspects of this effort may have value for both public and private conservation organizations. First, consistent with previous work (Coon et al., 2019; Smith et al., 2016), our results suggest that pumas may use developed landscapes as they travel between isolated habitat patches. Evaluating residency status might be achieved by conducting long‐term surveys on patches targeted for their connective value or conflict risk. The unsurveyed patches between the Hamilton Range and Mt Diablo serve in this capacity, whereas marshlands bordering the San Francisco Bay likely have little value for pumas as either habitat or stepping stones. In conjunction with Table 2, the thresholds we present here might be used as indices of extirpation vulnerability and for prioritizing crossing structures between patches with the greatest connective value (e.g., Burdett et al., 2010; Crooks et al., 2011). Second, small patch size and high edge‐area ratios can result in frequent conflict and high mortality (Benson et al., 2023; Woodroffe & Ginsberg, 1998). To reduce the potential for conflict associated with domestic animal depredation, isolated, but occupied patches should be targeted for public outreach and education activities (Vickers et al., 2015). Lastly, private lands are highly vulnerable to development but are critical for preserving the connectivity that still exists. To the extent possible, improved land‐use planning and permanent protection of suitable, connective habitat (Zeller et al., 2017) should be identified, mapped, and prioritized for targeted conservation.

## AUTHOR CONTRIBUTIONS

**David C. Stoner:** Conceptualization (lead); formal analysis (supporting); writing – original draft (lead); writing – review and editing (equal). **Zara McDonald:** Conceptualization (supporting); data curation (equal); funding acquisition (lead); investigation (equal); project administration (lead); writing – original draft (supporting); writing – review and editing (supporting). **Courtney A. C. Coon:** Conceptualization (equal); data curation (lead); formal analysis (lead); investigation (equal); methodology (lead); writing – original draft (equal); writing – review and editing (supporting).

## CONFLICT OF INTEREST STATEMENT

None.

## Data Availability

Data used in logistic regression models can be found in Table 2; all measured covariates will be made available from a publicly accessible repository upon publication.

## APPENDIX 1

**Table:**

Patch ID	Patch size (km^2^)	No. cameras in patch	Cameras per km^2^	Camera setup date	Data collection end date	Total camera trapping days	(cam*days)/km^2^
10	11.1	2	0.18	05‐08‐2020	22‐04‐2021	260	46.8
17	295.0	10	0.03	01‐02‐2020	24‐09‐2021	601	20.4
18	8.8	2	0.23	27‐06‐2020	05‐12‐2020	161	36.5
21	23.0	2	0.09	19‐02‐2021	12‐10‐2021	235	20.4
36	12.1	1	0.08	14‐12‐2017	28‐04‐2020	866	71.9
43	9.5	2	0.21	24‐06‐2020	21‐07‐2021	392	82.7
45	2.6	1	0.39	12‐08‐2020	05‐12‐2020	115	44.3
47	3.5	3	0.86	01‐11‐2020	01‐04‐2021	151	129.4

## APPENDIX 3

**Table:**

Model intercept	Med to high development	Fresh water	Grassland/agriculture	Patch size	Relative road length	df	logLik	AICc	Delta	Weight
−0.554	−17.27			0.50		3	−8.91	25.40	0.00	0.132
−2.592			2.75	0.44		3	−9.21	26.00	0.60	0.098
−1.227				0.43		2	−10.86	26.50	1.03	0.079
−1.785	−15.65		2.22	0.50		4	−8.00	26.90	1.43	0.065
1.088	−12.84					2	−11.18	27.10	1.68	0.057
−0.847			2.73			2	−11.33	27.40	1.98	0.049
−4.080		12.39	4.24	0.41		4	−8.29	27.40	2.01	0.048
0.065	−19.28				0.25	3	−10.01	27.60	2.19	0.044
−1.195	−20.30			0.45	0.17	4	−8.42	27.70	2.27	0.042
−2.622		13.76	4.36			3	−10.11	27.80	2.38	0.040
0.319						1	−12.93	28.10	2.67	0.035
0.004	−10.76		2.19			3	−10.27	28.10	2.72	0.034
−0.702	−16.79	1.90		0.48		4	−8.87	28.60	3.16	0.027
−1.542		4.53		0.40		3	−10.56	28.70	3.30	0.025
−1.290	−17.83		2.70		0.27	4	−9.10	29.10	3.63	0.021
−2.920			2.84	0.43	0.06	4	−9.16	29.20	3.75	0.020
−1.440				0.42	0.04	3	−10.82	29.20	3.82	0.020
−0.190		5.82				2	−12.40	29.60	4.13	0.017
0.701	−12.12	3.76				3	−10.98	29.60	4.14	0.017
−1.632			3.02		0.12	3	−11.03	29.70	4.23	0.016
−2.428	−18.50		2.32	0.46	0.16	5	−7.61	29.80	4.41	0.015
−2.973	−13.69	8.37	3.31	0.48		5	−7.61	29.80	4.41	0.015
−3.976		16.12	5.18		0.16	4	−9.56	30.00	4.55	0.014
−0.195					0.09	2	−12.72	30.20	4.77	0.012
−1.508	−8.01	10.20	3.36			4	−9.70	30.30	4.83	0.012
−0.488	−18.35	4.65			0.25	4	−9.74	30.30	4.90	0.011
−4.563		13.03	4.46	0.38	0.08	5	−8.18	31.00	5.55	0.008
−1.467	−19.82	3.02		0.43	0.18	5	−8.32	31.20	5.82	0.007
−1.034		6.94			0.12	3	−12.03	31.70	6.23	0.006
−1.960		5.14		0.39	0.07	4	−10.47	31.80	6.37	0.005
−2.747	−14.88	10.69	3.91		0.25	5	−8.59	31.80	6.38	0.005
−3.483	−16.50	8.34	3.33	0.43	0.15	6	−7.25	33.50	8.07	0.002
